# Squalamine and Its Derivatives Modulate the Aggregation of Amyloid-β and α-Synuclein and Suppress the Toxicity of Their Oligomers

**DOI:** 10.3389/fnins.2021.680026

**Published:** 2021-06-18

**Authors:** Ryan Limbocker, Roxine Staats, Sean Chia, Francesco S. Ruggeri, Benedetta Mannini, Catherine K. Xu, Michele Perni, Roberta Cascella, Alessandra Bigi, Liam R. Sasser, Natalie R. Block, Aidan K. Wright, Ryan P. Kreiser, Edward T. Custy, Georg Meisl, Silvia Errico, Johnny Habchi, Patrick Flagmeier, Tadas Kartanas, Jared E. Hollows, Lam T. Nguyen, Kathleen LeForte, Denise Barbut, Janet R. Kumita, Cristina Cecchi, Michael Zasloff, Tuomas P. J. Knowles, Christopher M. Dobson, Fabrizio Chiti, Michele Vendruscolo

**Affiliations:** ^1^Centre for Misfolding Diseases, Yusuf Hamied Department of Chemistry, University of Cambridge, Cambridge, United Kingdom; ^2^Department of Chemistry & Life Science, United States Military Academy, West Point, NY, United States; ^3^Laboratory of Organic Chemistry, Wageningen University, Wageningen, Netherlands; ^4^Laboratory of Physical Chemistry, Wageningen University, Wageningen, Netherlands; ^5^Department of Experimental and Clinical Biomedical Sciences, University of Florence, Florence, Italy; ^6^Enterin Inc., Philadelphia, PA, United States; ^7^MedStar Georgetown Transplant Institute, School of Medicine, Georgetown University, Washington, DC, United States; ^8^Cavendish Laboratory, Department of Physics, University of Cambridge, Cambridge, United Kingdom

**Keywords:** protein misfolding diseases, amyloid-β, Alzheimer’s disease, α-synuclein, Parkinson’s disease, oligomers, aminosterols, small molecule drug discovery

## Abstract

The aberrant aggregation of proteins is a key molecular event in the development and progression of a wide range of neurodegenerative disorders. We have shown previously that squalamine and trodusquemine, two natural products in the aminosterol class, can modulate the aggregation of the amyloid-β peptide (Aβ) and of α-synuclein (αS), which are associated with Alzheimer’s and Parkinson’s diseases. In this work, we expand our previous analyses to two squalamine derivatives, des-squalamine and α-squalamine, obtaining further insights into the mechanism by which aminosterols modulate Aβ and αS aggregation. We then characterize the ability of these small molecules to alter the physicochemical properties of stabilized oligomeric species *in vitro* and to suppress the toxicity of these aggregates to varying degrees toward human neuroblastoma cells. We found that, despite the fact that these aminosterols exert opposing effects on Aβ and αS aggregation under the conditions that we tested, the modifications that they induced to the toxicity of oligomers were similar. Our results indicate that the suppression of toxicity is mediated by the displacement of toxic oligomeric species from cellular membranes by the aminosterols. This study, thus, provides evidence that aminosterols could be rationally optimized in drug discovery programs to target oligomer toxicity in Alzheimer’s and Parkinson’s diseases.

## Introduction

Numerous neurodegenerative diseases are characterized by a complex pathophysiology that has made highly challenging the identification of targets for drug discovery and the development of clinical treatments (Cummings et al., 2014; Knowles et al., 2014; Chiti and Dobson, 2017). The aberrant misfolding, aggregation, and deposition of amyloid-β peptide (Aβ) and α-synuclein (αS) into pathological aggregates plays a central role in Alzheimer’s disease (AD) and Parkinson’s disease (PD), and recent evidence has demonstrated that oligomeric intermediates in the process of fibril formation are especially deleterious toward neuronal cells (Kreiser et al., 2020). These aggregates may, therefore, play a causative role in the onset and propagation of protein misfolding diseases throughout the central nervous system.

Much effort has been devoted toward understanding the ability of a variety of molecules to modify the aggregation reactions of these proteins (Dobson, 2003; Arosio et al., 2014; Knowles et al., 2014; Chiti and Dobson, 2017), resulting in the identification of species that enhance (Ladiwala et al., 2011; Lam et al., 2016; Limbocker et al., 2019) or reduce fibril formation (Ladiwala et al., 2011; Cohen et al., 2015; Habchi et al., 2016, 2017; Perni et al., 2017, 2018; Eskandari et al., 2020). Recent advances using chemical kinetics have enabled the characterization of numerous species that target specifically the microscopic steps governing protein aggregation, including primary nucleation, secondary nucleation, and elongation for Aβ (Cohen et al., 2015; Habchi et al., 2016, 2017) and lipid-induced nucleation, fibril amplification, and elongation for αS (Buell et al., 2014; Galvagnion et al., 2015; Flagmeier et al., 2016; Perni et al., 2018). Coupled with an analysis of the effects of the molecules on intermediate species *in vitro*, toward cells and after their administration to *Caenorhabditis elegans* expressing human αS or Aβ in their muscle cells, one can understand in detail the pathological consequences caused by perturbing the aggregation reaction with molecular species (Perni et al., 2017, 2018; Limbocker et al., 2019).

In addition to modulating the rates of oligomer formation, key studies have been carried out to identify the biophysical properties responsible for the oligomer-induced damage to cell membranes (Campioni et al., 2010; Bemporad and Chiti, 2012; Mannini et al., 2014; Cappelli et al., 2016; Chiti and Dobson, 2017; Fusco et al., 2017; Limbocker et al., 2020a). The relationship between oligomer size and hydrophobicity and their ability to embed and damage cell membranes provides insight into the origins of oligomer toxicity and illuminates a potential therapeutic avenue toward counteracting such toxicity. Specifically, it has been demonstrated that size and hydrophobicity are oppositely correlated to the ability of oligomers to damage cells, where small and hydrophobic oligomers are more toxic than larger and less hydrophobic ones (Mannini et al., 2014).

Aminosterols consist of a fused sterol linked to a polyamine side chain and are natural products originally discovered in the dogfish shark (Rao et al., 2000). As a class of naturally occurring molecules, aminosterols have been investigated for a host of medical applications ranging from obesity (Ahima et al., 2002), tissue regeneration (Smith et al., 2017), and cancer (Krishnan et al., 2014), to effective modulators of protein aggregation (Perni et al., 2017, 2018; Limbocker et al., 2019). Squalamine was first characterized for its ability to inhibit lipid-induced nucleation and to protect cell membranes by displacing oligomeric aggregates of αS at relatively low concentrations (Perni et al., 2017). Later on, we found that trodusquemine enhances the rate of aggregation of the 42-residue form of Aβ (Aβ_42_) by potentiating predominantly the secondary nucleation microscopic step (Limbocker et al., 2019) and inhibits αS aggregation by suppressing both the lipid-induced nucleation and fibril amplification processes (Perni et al., 2018). In both cases, this molecule could reduce the lifetime or number of oligomeric species by catalyzing their conversion to the fibrillar form for Aβ_42_ (Limbocker et al., 2019) or attenuating their rate of formation for αS (Perni et al., 2018). Trodusquemine prevents the toxicity of isolated or stabilized oligomers comprised of the 40-residue form of Aβ (Aβ_40_), Aβ_42_, αS, and the model protein HypF-N by binding to the cell membrane (Errico et al., 2020), outcompeting oligomers at the cell membrane, and therein displacing these aggregates from cell membranes through a conserved mechanism (Perni et al., 2018; Limbocker et al., 2019, 2020b). Trodusquemine was found not to directly target either the size or hydrophobicity of the oligomers at physiological concentrations, but instead to function through a mechanism based on oligomer displacement from the cell membrane in addition to its ability to modulate the kinetics of their assembly (Perni et al., 2018; Limbocker et al., 2019, 2020b). Finally, squalamine was recently reported to prevent the aggregation of αS and its associated toxicity in new *C. elegans* models of familial forms of PD, where its beneficial effect was more pronounced in A53T worms than in the A30P ones (Perni et al., 2021).

In this report, we sought to resolve the effects of different aminosterols, including trodusquemine (TRO), squalamine (SQ), α-squalamine (αSQ), and des-squalamine (desSQ), on the aggregation reactions of Aβ and αS. By investigating the kinetics of oligomer formation, the structural properties of oligomers upon aminosterol addition at both physiological and molar excess concentrations, and using tissue culture experiments, we demonstrate that subtle structural differences in these aminosterols can lead to differential efficacies in preventing the toxicity linked to protein aggregation. In addition to providing a framework to characterize relevant molecules, these results further signal the relevance of aminosterols in the treatment of protein misfolding diseases and suggest that future studies to optimize aminosterol potency and efficacy may be possible.

## Materials and Methods

### Reagents

Squalamine and its derivatives were synthesized as a dilactate, and TRO as a hydrochloride salt at purity greater than 97% and stored as a lyophilized powder. Aliquots were prepared at 10 mM in water and stored at −20°C.

### Preparation of Aβ_42_ for Chemical Kinetics Experiments

The recombinant Aβ(M1–42) peptide (MDAEFRHDSGY EVHHQKLVFF AEDVGSNKGA IIGLMVGGVV IA), denoted Aβ_42_, was expressed in the *Escherichia coli* BL21 Gold (DE3) strain (Stratagene, San Diego, CA, United States) and purified as described previously (Habchi et al., 2017). The recombinant Aβ(M1–40) peptide (MDAEFRHDSGY EVHHQKLVFF AEDVGSNKGA IIGLMVGGVV), denoted Aβ_40_, was similarly prepared. Samples were prepared for kinetic experiments using standard reagents and methods. Briefly, monomeric protein was purified in 20 mM sodium phosphate buffer at pH 8 supplemented with 200 μM EDTA (Habchi et al., 2017). Thioflavin-T (ThT, Sigma-Aldrich, St. Louis, MO, United States) was added from a 1 mM stock to a final concentration of 20 μM. All samples were prepared in low-binding Eppendorf tubes, and samples were analyzed in a 96-well half area, low-binding, clear-bottom PEG-coated plate (Corning 3881, Sigma-Aldrich, St. Louis, MO, United States). Fibril seeds were prepared as described previously (Habchi et al., 2017).

Concentrations of ThT of 20 μM or below have been described to exert minimal effects on aggregation kinetics experiments (Xue et al., 2017). The effects of aminosterols have been previously characterized not to be related to aminosterols binding artifactually to ThT rather than amyloid aggregates (Perni et al., 2018). Previous work used ThT to monitor the aggregation kinetics of Aβ_42_ in the absence and presence of the primary nucleation inhibitor bexarotene. The degree of inhibition, as quantified with the ThT experiments, corresponded directly to AFM and dot-blot measurements taken in the absence of ThT both in the absence and presence of a fourfold excess of bexarotene (Habchi et al., 2016), indicating that ThT does not appear to alter the aggregation reaction both with and without bexarotene under these conditions. While we cannot completely exclude that ThT has some effect on the mechanisms of the Aβ_42_ and αS aggregation reactions, it is unlikely that the described effects of the aminosterols are related to ThT-based artifacts.

### Aβ_42_ Kinetic Analysis

ThT fluorescence was monitored in triplicate per sample as measured using the bottom optic in a plate reader (Fluostar Omega or Fluostar Optima from BMGLabtech, Aylesbury, United Kingdom) with 440- and 480-nm excitation and emission filters, respectively. Aggregation was initiated by transferring the 96-well plate to the plate reader at 37°C under quiescent conditions. The time evolution of the total fibril mass concentration, *M*(*t*) (Cohen et al., 2012, 2013), was analyzed as described in the main text.

### αS Expression and Lipid Preparation

Wild-type human αS was recombinantly expressed and purified as described previously (Hoyer et al., 2002; Galvagnion et al., 2016). For concentration measurements, an extinction coefficient of 5,600 M^–1^ cm^–1^ was used at 275 nm. After the final size exclusion chromatography (phosphate buffer, pH 6.5, 20 mM), the protein was snap frozen in liquid nitrogen in the form of 1-ml aliquots and stored at −80°C. The lipids were dissolved in 20 mM phosphate buffer (NaH_2_PO_4_/Na_2_HPO_4_), pH 6.5, 0.01% NaN_3_, and stirred at ca. 45°C for 2 h. The solution was then frozen and thawed five times using dry ice and a water bath at 45°C. The preparation of vesicles was carried out using sonication at 3 × 5 min, 50% cycles, 10% maximum power on ice with a Bandelin Sonopuls HD 2070 (Bandelin, Berlin, Germany). After centrifugation, the sizes of the vesicles were checked using dynamic light scattering (Zetasizer Nano ZSP, Malvern Instruments, Malvern, United Kingdom) and were shown to consist of a distribution centered at a diameter of 20 nm.

### Aggregation Kinetics of αS in the Presence of Lipid Vesicles

1,2-Dimyristoyl-sn-glycero-3-phospho-L-serine (sodium salt; DMPS) was purchased from Avanti Polar Lipids (Alabaster, AL, United States). αS was incubated at a concentration of 100 μM in 20 mM sodium phosphate, pH 6.5, 0.01% NaN_3_, in the presence of 50 μM ThT, 100 μM DMPS vesicles, and increasing concentrations of aminosterols (0–10 μM). The stock solution of each aminosterol was prepared by dissolving the molecule in 20 mM phosphate buffer to a final concentration of 100 μM. The change in the ThT fluorescence signal with time was monitored using a Fluostar Optima or a Polarstar Omega (BMG Labtech, Aylesbury, United Kingdom) under quiescent conditions at 30°C (Perni et al., 2017) in the above-described Corning 3881 96-well plates.

### Atomic Force Microscopy of Fibrils

Aβ_42_ fibrils were prepared by incubating the protein from its monomeric state (2 μM) for 4 h in the absence or presence of an equimolar concentration of SQ (2 μM) in the absence of ThT. The incubation time of 4 h was selected as this time is sufficient for all samples to enter the plateau phase from ThT kinetics experiments. Mica substrates were positively functionalized by their incubation with a 10-μl drop of 0.05% (v/v) (3-aminopropyl)triethoxysilane (APTES, Sigma-Aldrich, St. Louis, MO, United States) in Milli-Q water for 1 min at ambient temperature, followed by rinsing with Milli-Q water and gentle drying with gaseous nitrogen (Ruggeri et al., 2015, 2016). AFM sample deposition was carried out at room temperature by depositing a 10-μl drop of protein at a concentration of 2 μM for 2 min. to a mica surface treated with APTES. Salt was washed with high-purity water, and samples were stored in a sealed container until imaging using a JPK Nanowizard2 system (JPK Instruments, Berlin, Germany) using tapping mode with scan rates <0.5 Hz and a silicon tip with a 10-nm nominal radius (MikroMasch, Wetzlar, Germany).

### Transmission Emission Microscopy

Samples for TEM were prepared on a 400-mesh, 3-mm copper grid carbon support film (EM Resolutions Ltd., Sheffield, United Kingdom) and stained with 2% uranyl acetate (wt/vol) (Limbocker et al., 2019). The samples were imaged on a FEI Tecnai G2 transmission electron microscope (Cambridge Advanced Imaging Centre, CAIC, University of Cambridge, United Kingdom). Images were acquired using the SIS Megaview II Image Capture system (Olympus, Muenster, Germany).

### αS Oligomer Preparation

αS was prepared as previously described (Chen et al., 2015). Briefly, protein was purified into PBS, and subsequently dialyzed against water (4 L, overnight at 4°C). Six-milligram aliquots were lyophilized for 2 days, followed by resuspension in buffer (500 μl of 20 mM Tris, 100 mM NaCl, pH 7.4). The resuspended protein was passed through 0.22-μm filters and incubated (20–24 h, 37°C). The samples were ultracentrifuged (1 h, 90,000 rpm, 20°C) in a TLA120.2 rotor, using an Optima TLX Ultracentrifuge (both Beckman Coulter, High Wycombe, United Kingdom) to remove aggregates and large oligomers. The remaining monomer was removed using a 100-kDa centrifugation filter (4×) (2 min, 10,000 rpm). The flow through containing predominantly monomer from the first three passes was kept and reused up to five times. Oligomer concentration was determined by UV spectroscopy, using an extinction coefficient of 5,600 M^–1^cm^–1^ at a wavelength of 275 nm.

### Aβ_40_ Oligomer Preparation

All samples were prepared using LoBind tubes. Lyophilized Aβ_40_ (0.5 or 1.0 mg) was solubilized to a monomeric form overnight in 300 μl of HFIP. The following day, the solvent was gently evaporated off with nitrogen, and the protein resuspended in 100% DMSO with thorough pipetting at a final concentration of 2.2 mM. Two sonication steps of 10 min were preformed, then protein was resuspended at 100 μM in 20 mM sodium phosphate buffer, 200 μM ZnCl_2_, pH 6.9 (Mannini et al., 2018). After 20 h at 20°C, samples were spun down at 15,000 rpm at 20°C for 15 min. The supernatant was removed, and the pellet containing the oligomers was resuspended in buffer (20 mM Tris, 100 mM NaCl at pH 7.4). While Aβ_42_ is generally more toxic than Aβ_40_, it is challenging to carry out a comprehensive biophysical characterization of Aβ_42_ oligomers as they are difficult to prepare in a stable, homogeneous population in near-physiological conditions (Kulenkampff et al., 2021). As such, we elected to investigate oligomers of Aβ_40_ stabilized by zinc ions (Zn^2+^), which can be generated at near-physiological conditions in a stable, homogenous population. Moreover, Aβ_40_ comprises a majority of the insoluble amyloidogenic plaques in the AD brain and is, therefore, an important oligomeric agent in disease (Mannini et al., 2018).

### HypF-N Oligomer Preparation

HypF-N was purified as previously reported (Campioni et al., 2010; Capitini et al., 2018). Type A toxic oligomers were prepared as previously described (Campioni et al., 2010). Briefly, purified wild-type monomeric HypF-N was resuspended at 0.5 mg/ml in 12% TFE, 2 mM DTT, 50 mM acetate buffer, pH 5.5 to a total volume of 500 μl and incubated in a water bath at 25°C. After 4 h of incubation, protein was spun down at 16,100 × *g* at 20°C for 15 min. The supernatant was removed, and residual solvent was evaporated off by gently drying the pellet with nitrogen gas, and the pellet containing the oligomers was resuspended in buffer (20 mM Tris, 100 mM NaCl at pH 7.4).

### Preparation of Aβ-Derived Diffusible Ligand Oligomers of Aβ_42_

Lyophilized Aβ_42_ (Sigma-Aldrich, St. Louis, MO, United States) was dissolved in 100% hexafluoro-2-isopropanol (HFIP) to 1.0 mM, and then the solvent was evaporated. Aβ-derived diffusible ligands of Aβ_42_ (ADDLs) oligomers were then prepared according to the Lambert’s protocol (Lambert et al., 2001).

### Aminosterol Incubation With Oligomers

After oligomer formation, samples were incubated in the absence or presence of aminosterols for 1 h at 20°C in 20 mM Tris, 100 mM NaCl at pH 7.4. Samples were prepared at a final concentration of 5 μM oligomers, and aminosterols were added at 1-, 2. 5-, 5-, 7. 5-, and 10-fold excesses, unless otherwise stated.

### 8-Anilinonaphthalene-1-Sulfonate Binding Measurements

Solutions with oligomers at a concentration of 5 μM in buffer (20 mM Tris, 100 mM NaCl at pH 7.4) were aliquoted, and 8-anilinonaphthalene-1-sulfonate (ANS) was added to a final concentration of 15 μM from a concentrated stock. Emission spectra were recorded using a plate reader (BMG Labtech, Aylesbury, United Kingdom) with excitation at 380 nm. Spectra were background subtracted to that of the spectra of buffer alone.

### Turbidity Measurements

Samples from the ANS preparation were analyzed using a plate reader (BMG Labtech, Aylesbury, United Kingdom) with spectral scanning. Spectra were background subtracted to that of the spectra of buffer alone.

### Atomic Force Microscopy of Oligomers

Oligomers were incubated at 5 μM in 20 mM Tris, 100 mM NaCl at pH 7.4 in the absence or presence of 25 μM SQ. All samples were diluted by a factor of five and subsequently sprayed at 100 μl/h for 1 min at room temperature using a recently described microfluidic device (Ruggeri et al., 2018) atop an atomically flat MICA surface. AFM measurements were performed by using a Park NX10 AFM (Park Systems, Suwon, South Korea) with scan rates <0.3 Hz and PPP-NCHR cantilevers with an 8-nm nominal radius (Nanosensors, Neuchatel, Switzerland).

### Neuroblastoma Cell Culture

Human SH-SY5Y neuroblastoma cells (ATCC, Manassas, VA, United States) were cultured in DMEM, F-12 HAM with 25 mM HEPES, and NaHCO_3_ (1:1) and supplemented with 10% FBS, 1 mM glutamine, and 1.0% antibiotics. Cell cultures were maintained in a 5% CO_2_ humidified atmosphere at 37°C and grown until they reached 80% confluence for a maximum of 20 passages (Cascella et al., 2019). The cell line was authenticated and tested negative for mycoplasma contamination.

### MTT Reduction Assay

Zn^2+^-stabilized Aβ_40_ (Mannini et al., 2018) (5 μM, in monomer equivalents), HypF-N (Campioni et al., 2010) (6 μM, in monomer equivalents), and ADDLs of Aβ_42_ oligomers (Lambert et al., 2001) (1 μM, in monomer equivalents) were incubated with or without increasing concentrations of SQ for 1 h at 37°C under shaking conditions, and then added to the cell culture medium of SH-SY5Y cells seeded in 96-well plates for 24 h. The 3-(4,5-dimethylthiazol-2-yl)-2,5-diphenyltetrazolium bromide (MTT) reduction assay was performed as previously described (Cascella et al., 2017).

### Oligomer Binding to the Cellular Membrane

SH-SY5Y cells were seeded on glass coverslips and treated for 15 min with oligomers of various proteins at the listed concentrations in the absence or presence of SQ. After incubation, the cells were washed with PBS and counterstained with 5.0 μg/ml of Alexa Fluor 633-conjugated wheat germ agglutinin (Life Technologies, CA, United States) (Perni et al., 2017). After washing with PBS, the presence of oligomers was detected with 1:800 diluted mouse monoclonal 6E10 anti-Aβ antibodies (BioLegend, San Diego, CA, United States) or 1:800 rabbit anti-HypF-N antibodies (Primm, Milan, Italy) and subsequently with 1:1,000 diluted Alexa Fluor 488-conjugated anti-mouse or anti-rabbit secondary antibodies (Life Technologies, CA, United States). Fluorescence emission was detected after double excitation at 488 and 633 nm by a scanning confocal microscopy system (Perni et al., 2017), and three apical sections were projected as a single composite image by superimposition. ImageJ (NIH, Bethesda, MD, United States) and JACOP plugin (rsb.info.nih.gov) software were used to calculate the percentage of colocalization between cell membranes and oligomers.

### Statistics

Data were analyzed using GraphPad Prism 8 (CA, United States) using an unpaired, two-tailed Student’s *t*-test or one-way ANOVA followed by Bonferroni’s post comparison test relative to cells treated with culture media or oligomers, as indicated in the corresponding figure legends.

## Results

**FIGURE 1:**
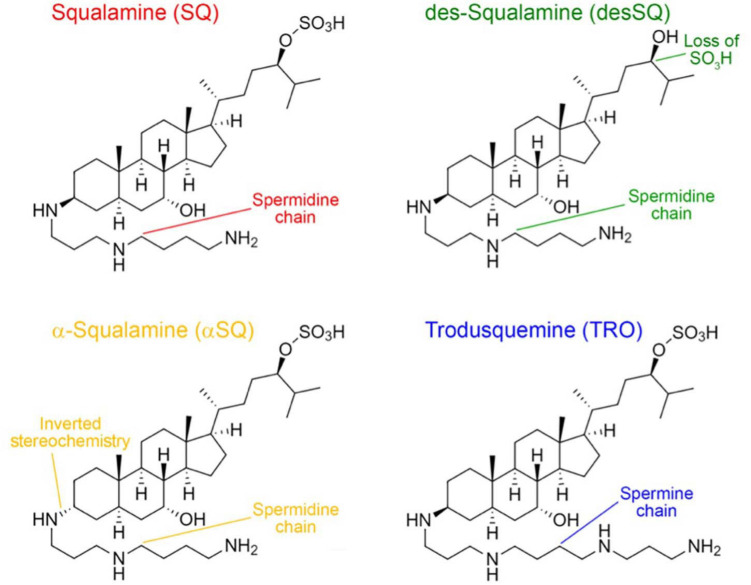
Structures of the four aminosterols investigated in this work. Squalamine (SQ, red), des-squalamine (desSQ, green), α-squalamine (αSQ, orange), and trodusquemine (TRO, blue) with unique molecular features indicated.

In this work, we analyzed four aminosterols, squalamine (SQ), des-squalamine (desSQ), α-squalamine (αSQ), and trodusquemine (TRO), with slightly different physicochemical properties (Figure 1). The investigation of the addition of polyamine groups to a sterol moiety is particularly interesting following recent reports about the remarkable ability of polyamines to modulate cellular homeostasis (Schroeder et al., 2021; Liang et al., 2021). TRO contains a spermine moiety as its polyamine side chain, while SQ and its derivatives have a spermidine linked to the fused sterol ring. Within the SQ derivatives, αSQ has inverted stereochemistry about the carbon atom which links the spermidine to the sterol, while desSQ lacks the SO_4_H group on its alkyl chain (Figure 1). At neutral pH, TRO has a net charge of +3. SQ and αSQ bear one less positive charge relative to TRO on its side chain, and SQ was initially discovered in the dogfish shark liver at a ratio of SQ to TRO of 99:1 (Rao et al., 2000). DesSQ has the same net positive charge relative to TRO. We sought to determine if either inverting the stereochemistry or removing the SO_3_H group to increase its overall charge by one, as occurring in αSQ and desSQ relative to SQ, respectively, would impact the efficacy of aminosterols in modulating the rate of αS and Aβ protein aggregation or their oligomer binding to cell membranes.

### Effects of the Aminosterols on the Aggregation Kinetics of Aβ_42_ and αS

The kinetics underpinning protein aggregation play a key role in the production of oligomeric species, which are highly toxic to cells (Chia et al., 2018; Michaels et al., 2020). A great deal of work has been carried out to determine the effect of additive agents to the aggregation of the Aβ peptide, including small molecules (Habchi et al., 2016, 2017, 2018; Chia et al., 2018; Limbocker et al., 2019), molecular chaperones (Cohen et al., 2015), and antibodies (Aprile et al., 2017; Limbocker et al., 2020a). Recent work has shown that one can directly link the number of oligomers produced during an aggregation reaction to the kinetics of their fibrilization, where inhibitors decrease the number of oligomers formed (Chia et al., 2018; Michaels et al., 2020).

**FIGURE 2:**
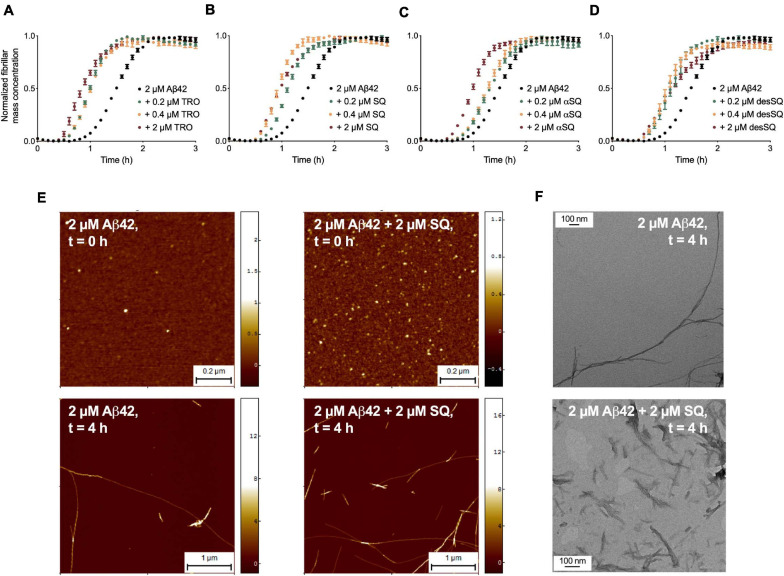
Aminosterols accelerate Aβ_42_ aggregation *in vitro*. Kinetic profiles of the aggregation of 2 μM Aβ_42_ in the absence (black) or presence of 0.2 μM (green), 0.4 μM (orange), and 2 μM (red) of TRO **(A)**, SQ **(B)**, αSQ **(C)**, and desSQ **(D)**. Data represent mean ± standard error of the mean (s.e.m.) of three technical replicates. Raw ThT aggregation data are provided in Supplementary Figure 1. **(E)** AFM images at *t* = 0 h (top panels, scale bars = 0.2 μm) and *t* = 4 h (bottom panels, scale bars = 1 μm) of aggregation in the absence (left panels) and presence of an equimolar concentration of SQ (right panels). **(F)** TEM images of fibrils formed after 4 h of aggregation in the absence (top) or presence of an equimolar concentration of SQ (bottom). Scale bars = 100 nm. AFM and TEM measurements were taken alongside a comparison of the effect of TRO on Aβ_42_ aggregation, and the untreated fibrils, therefore, have the same population statistics as previously published (Limbocker et al., 2019).

We began this study by probing the effects of the aminosterol derivatives on the aggregation of Aβ_42_. Past work has demonstrated that TRO enhances the rate of secondary nucleation in Aβ_42_ aggregation (Limbocker et al., 2019) and reduces the rates of lipid-induced nucleation and fibril amplification in αS aggregation (Perni et al., 2018). We first carried out unseeded aggregation assays *in vitro* in the presence of thioflavin-T (ThT) as described previously for Aβ_42_ (Habchi et al., 2017) to evaluate the effects of aminosterols on the aggregation processes of this protein. The aggregation of 2 μM Aβ_42_ was monitored in the absence and presence of increasing concentrations of each aminosterol (0.2, 0.4, and 2 μM) (Figures 2A–D and Supplementary Figure 1). As the aggregation reaction for Aβ_42_ plateaus upon monomer depletion, we normalized each condition to the plateau phase of the ThT trace to obtain the normalized fibril mass concentration over time in Figures 2A–D, while the raw ThT data can be found in Supplementary Figure 1. In the presence of all these compounds, Aβ_42_ aggregation was enhanced.

We next sought to assess the effects of the aminosterols on further processes governing Aβ_42_ aggregation, namely, secondary nucleation and elongation (Habchi et al., 2017; Limbocker et al., 2019). In light of the similarity in behavior between the aminosterols in the unseeded aggregation assays, we elected to quantify only the effects of SQ to facilitate a comparison with TRO that was published previously (Limbocker et al., 2019). Seeded aggregation assays with Aβ_42_ at a concentration of 2 μM were carried out in the presence of a low concentration of fibril seeds (i.e., 5% fibrils, in monomer equivalents), where primary nucleation is not rate limiting, and with a high concentration of fibril seeds (i.e., 25% fibrils, in monomer equivalents), where elongation is the rate-determining step, in order to monitor the effects of SQ on the microscopic rate constants associated with the aggregation of Aβ_42_ (Habchi et al., 2017).

The aggregation of 2 μM Aβ_42_ was carried out in the unseeded aggregation assay (Supplementary Figure 2a) and in the presence of 5% preformed seed fibrils (Supplementary Figure 2b), which caused a characteristic reduction in the half-time of aggregation. Moreover, upon the addition of increasing concentrations of SQ (0.2, 0.4, and 2 μM), the half-times were further reduced (Supplementary Figure 2b). As in this environment primary nucleation is not rate limiting, the observed increase in aggregation is likely to be associated with secondary nucleation pathways. We then followed the aggregation of 2 μM Aβ_42_ in the presence of 25% preformed seeds, which caused a dramatic reduction in the half-time of aggregation. Again, the presence of increasing concentrations of SQ (0.2, 0.4, and 2 μM) further reduced the half-time of aggregation (Supplementary Figure 2c), but this enhancement of aggregation was small in comparison with the unseeded and 5% seeded assays.

The time evolution of the total fibril mass concentration, *M*(*t*), was described by an integrated rate law containing combinations of the microscopic rate constants *k*_+_*k*_2_ and *k_*n*_k_2_*, where *k*_*n*_, *k*_+_, and *k*_2_ indicate primary nucleation, elongation, and secondary nucleation rate constants, respectively (Cohen et al., 2012, 2013). The perturbation induced by SQ was then resolved by fitting experimental data to a master equation to identify the alterations to the microscopic processes, as described in detail previously (Habchi et al., 2017). Our results reveal that the unseeded and 5% seeded data are well-described by varying the rates associated with secondary nucleation pathways, *k*_+_*k*_2_, or secondary nucleation, k2, respectively (Supplementary Figures 2a,b, solid lines). Furthermore, fitting the unseeded and 5% seeded data for an effect on *k*_2_ matched the experimental data well (Supplementary Figure 2d, solid lines), while fitting for primary nucleation events, *k*_*n*_, was less accurate (Supplementary Figure 2e, solid lines). Moreover, the seeded data suggest a weak increase in elongation and a strong increase in secondary nucleation. Indeed, the 5% seeded data are aptly described by varying only the rate associated with secondary nucleation (Supplementary Figure 2b, solid lines), and the rate of elongation alone is insufficient to recapitulate the overall observed increase in aggregation in all three of these assays. These results are comparable with those previously described for TRO (Limbocker et al., 2019) and suggest that these four aminosterols exert comparable accelerating effects on secondary processes governing the Aβ_42_ aggregation reaction.

To validate the kinetic model and visualize the morphology of the aggregates formed in the presence of aminosterols, we carried out high-resolution and phase-controlled non-contact atomic force microscopy (AFM). Fibrils were quantified after incubation for 4 h in the absence and presence of an equimolar concentration of SQ and in the absence of ThT by measuring both their lengths and cross-sectional heights, where the latter feature corresponds to the diameter of the aggregates (Figure 2E). We found that fibril height was increased significantly in the presence of SQ, from 4.2 ± 0.2 nm [mean ± total error, as calculated in Limbocker et al. (2019); *n* = 387] in its absence to 5.8 ± 0.4 nm (*n* = 138) in its presence (*p* < 0.001 by a paired *t*-test). Additionally, the average fibril length was observed to decrease in the presence of SQ, from 1.81 ± 0.12 μm (*n* = 100) in its absence to 0.60 ± 0.06 μm (*n* = 100) in its presence (*p* < 0.001 by a paired *t*-test), and transmission emission microscopy images confirmed the reduction in fibril length by SQ (Figure 2F). The fibril morphology is, therefore, in agreement with the expectation that an increase in secondary nucleation would shift the aggregation reaction toward the formation of shorter fibrils. The resulting kinetic and morphological measurements, therefore, indicate that TRO and SQ modulate the assembly of Aβ_42_ in a very similar manner by stimulating secondary nucleation.

**FIGURE 3:**
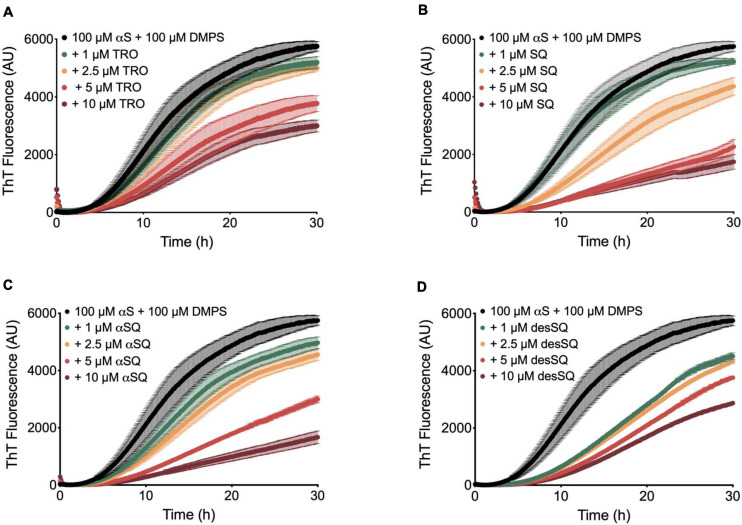
Aminosterols inhibit the lipid-induced nucleation of αS. Kinetic profiles of the aggregation of 100 μM αS with 100 μM 1,2-dimyristoyl-sn-glycero-3-phospho-L-serine (DMPS) vesicles in the absence (black) or presence of 1 (green), 2.5 (orange), 5 (red), and 10 (dark red) μM concentrations of TRO **(A)**, SQ **(B)**, αSQ **(C)**, and desSQ **(D)**. Data represent mean ± s.e.m. of three technical replicates.

We then quantified the effects of the aminosterol derivatives on αS aggregation. In a similar manner as previously described (Perni et al., 2017), the lipid-induced aggregation of 100 μM monomeric αS with 100 μM DMPS vesicles was monitored over time using ThT at a concentration of 50 μM (20 mM phosphate buffer, pH 6.5, 0.01% NaN_3_) in the absence and presence of increasing concentrations of aminosterols (1–10 μM) (Figure 3). In agreement with previous findings for SQ (Perni et al., 2017), we observe that the molecules inhibited the rate of lipid-induced nucleation and reduced the quantity of ThT-sensitive aggregates that formed during the aggregation process. We note that these conditions are used for monitoring lipid-induced nucleation in αS aggregation, whereas fibril elongation or secondary nucleation can be studied specifically by changing the solution conditions through the addition of fibril seeds or acidification of the aggregation reaction, respectively (Buell et al., 2014). The effects observed herein for aminosterols are, therefore, likely related to the initiation steps in αS aggregation, rather than secondary processes. As described previously, SQ displaces monomeric αS from the vesicle surfaces, thereby blocking the first steps in its aggregation process (Perni et al., 2017).

**FIGURE 4:**
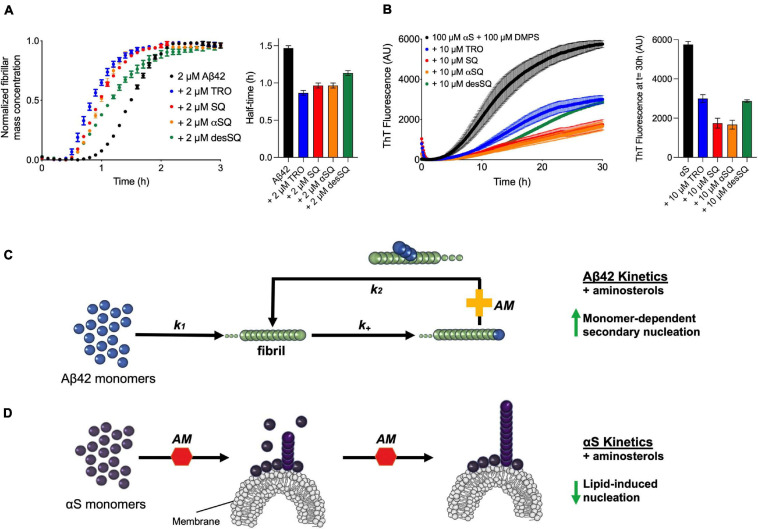
Aminosterols modify the aggregation processes of Aβ_42_ and αS. **(A)** Kinetic profiles of the aggregation of 2 μM Aβ_42_ in the absence (black) or presence of an equimolar concentration of each aminosterol (represented with various colors). The bar plot shows the half-time of Aβ_42_ aggregation in the absence or presence of the aminosterols. **(B)** Kinetic profiles of the aggregation of 100 μM αS in the absence (black) or presence of 10 μM of each aminosterol (represented with various colors). Since trodusquemine has been characterized not to quench ThT or self-assemble into larger species under these conditions (Perni et al., 2018), the decrease in ThT signal for the aminosterols studied is not likely related to ThT artifacts or assemblies of TRO, such as micelles. The bar plot shows the ThT signal quantified after 30 h of αS aggregation in the absence or presence of the aminosterols. In panels **(A–D)**, data represent mean ± s.e.m. of three technical replicates and are the kinetic traces reproduced from Figures 2, 3. **(C)** Schematic illustration of the effect of aminosterols (AM) on Aβ_42_ aggregation, where they enhance monomer-dependent secondary nucleation (*k*_2_). **(D)** Schematic illustration of the effect of aminosterols on αS aggregation, where they inhibit lipid-induced primary nucleation.

We finally compared the effects of these four aminosterols for both aggregation reactions and found the results to be highly similar, where Aβ_42_ was enhanced and αS inhibited to comparable extents, as shown by comparing the aggregation traces in the presence of either one molar equivalent of each aminosterol in the presence of Aβ_42_ (Figure 4A) or 10 μM of each molecule for αS (Figure 4B), illustrating that aminosterols enhance the monomer-dependent secondary nucleation microscopic step in Aβ_42_ aggregation (Figure 4C) and inhibit the lipid-induced primary nucleation process in αS aggregation (Figure 4D).

### Aminosterol Derivatives Modulate the Size–Hydrophobicity Relationship of Stabilized Oligomers

After resolving the kinetics associated with oligomer formation for the aggregation reactions of αS and Aβ_42_ in the presence of aminosterols, we next sought to characterize the effects of these molecules on oligomeric aggregates of αS (Chen et al., 2015) and Aβ_40_ stabilized by Zn^2+^ ions (Mannini et al., 2018). These model species may represent the toxic aggregates observed in pathology due to their formation protocol or stabilization by a metal ion naturally present at the synaptic cleft, respectively. In doing so, we sought to elucidate if all the aminosterols studied here could modulate the size or hydrophobicity of these stabilized oligomers, given that these properties have been demonstrated to be key mediators of oligomer toxicity (Mannini et al., 2014; Limbocker et al., 2020a). TRO was previously shown to function at concentrations at and below molar equivalence with respect to oligomers to prevent oligomer binding and toxicity, and these concentrations did not significantly change the structural properties of oligomers that play a central role in their ability to induce dysfunction (Limbocker et al., 2020b). Higher concentrations of TRO in excess of its physiological range and rationally designed antibodies, however, have been shown to increase the size and hydrophobicity of Zn^2+^-stabilized Aβ_40_ oligomers (Limbocker et al., 2020a). In the experiments with antibodies, both the size and hydrophobicity were increased in a similar fashion and with an approximately linear relationship, which would be expected to cause an offsetting decrease and increase in oligomer toxicity, respectively. Experimentally, Aβ_40_ oligomer toxicity did not change after incubation with the designed antibodies (Limbocker et al., 2020a). In the experiments with TRO, both the size and hydrophobicity were also increased in a similar fashion and approximately linear relationship, but toxicity was not tested in this case due to the inherent toxicity of TRO to cells above a concentration of circa 6 μM, where these physiochemical modifications were observed for the oligomers (Limbocker et al., 2020a).

**FIGURE 5:**
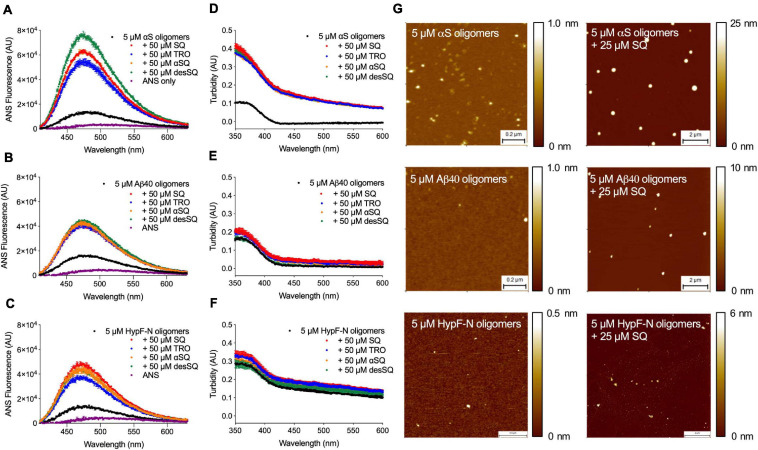
Aminosterols modify the hydrophobicity and size of αS, Aβ_40_ stabilized by Zn^2+^, and HypF-N oligomers. **(A–F)** 8-Anilinonaphthalene-1-sulfonate (ANS) binding measurements to probe the hydrophobicity of oligomers (5 μM in monomer equivalents) of αS **(A)**, Aβ_40_ **(B)**, and HypF-N **(C)** with corresponding size changes monitored by turbidity absorbance **(D–F)** in the absence and presence of a 10-fold excess of SQ, TRO, αSQ, and desSQ (represented in different colors). ANS alone is shown for reference in **(A–C)** (purple). Data represent mean ± s.e.m. of two technical replicates. Dose-dependent measurements for ANS fluorescence and turbidity absorbance can be found in Supplementary Figures 3–5 ranging from 0 to 50 μM of each aminosterol. A 50 μM concentration of each aminosterol in the absence of oligomers did not noticeably impact ANS fluorescence or turbidity absorbance relative to the effect observed for oligomers and the respective concentrations of each molecule (Supplementary Figures 3–5). **(G)** The indicated oligomers in the absence and presence of a fivefold excess of SQ visualized using high-resolution, phase-controlled AFM after sample preparation using a microfluidic device (Ruggeri et al., 2018).

In addition to studying the pathologically linked oligomers of αS and Aβ_40_, we also investigated toxic oligomers of HypF-N, an *E. coli* protein not associated with human pathology, as a generic model of misfolded protein oligomers (Campioni et al., 2010). After oligomer formation, solutions containing the molecules were incubated for 1 h at 20°C in the absence or presence of increasing concentrations of the four aminosterols (0–50 μM), after which time 15 μM 8-anilinonaphthalene-1-sulfonate (ANS) was added to probe the hydrophobicity of the oligomers. The same samples were also analyzed for their turbidity absorbance using a plate reader. We observed that the hydrophobicity (Figures 5A–C and Supplementary Figures 3–5) and size (Figures 5D–F and Supplementary Figures 3–5) of the oligomers were augmented in the presence of increasing concentrations of all four aminosterols for oligomers of αS, Aβ_40_, and HypF-N as measured using ANS binding and absorbance, in agreement with our previous characterization of Aβ_40_ oligomers and TRO (Limbocker et al., 2020a). In these assays and at molar excess concentrations, the aminosterol derivatives exhibited similar behaviors (Figure 5 and Supplementary Figures 3–5). For all oligomer systems and as previously described for high concentrations of TRO and specific rationally designed antibodies (Limbocker et al., 2020a) with Zn^2+^-stabilized Aβ_40_ oligomers, a concomitant increase in size and hydrophobicity was observed with increasing concentrations of aminosterols. These effects would be expected not to change the toxicity of the oligomers, as previously described in detail (Limbocker et al., 2020a), as typically an increase in oligomer size is protective against oligomer toxicity, while an increase in their hydrophobicity is deleterious.

To further characterize the size of the aggregates in the absence of ANS, we performed high-resolution, phase-controlled AFM using a recently developed microfluidic spraying platform (Ruggeri et al., 2018). This technology circumvents mass-transport processes that can lead to artifacts resulting from the drying process of an aqueous solution during conventional AFM sample preparation processes, as the device sprays sub-picoliter volumes that dry nearly instantaneously on the mica substrate (Ruggeri et al., 2018). Given the similarity in the bulk oligomer measurements (Figures 5A–F) and aggregation experiments (Figures 2–4), we elected to further investigate only SQ in detail. Oligomers were incubated as previously described in the absence or presence of a fivefold excess concentration of SQ and diluted in buffer by a factor of five prior to deposition atop a mica substrate for AFM imaging. When quantified by AFM, oligomers typically have a cross-sectional height of 2–6 nm for HypF-N (Campioni et al., 2010), 1–5 nm for Zn^2+^-stabilized Aβ40, and 3–16 nm for αS (Chen et al., 2015). The presence of significantly larger aggregates was observed in the presence of SQ for all three oligomer systems (Figure 5G), in direct agreement with the turbidity data.

### Squalamine Attenuates the Toxicity of Oligomers by Displacing Them From Cell Membranes

**FIGURE 6:**
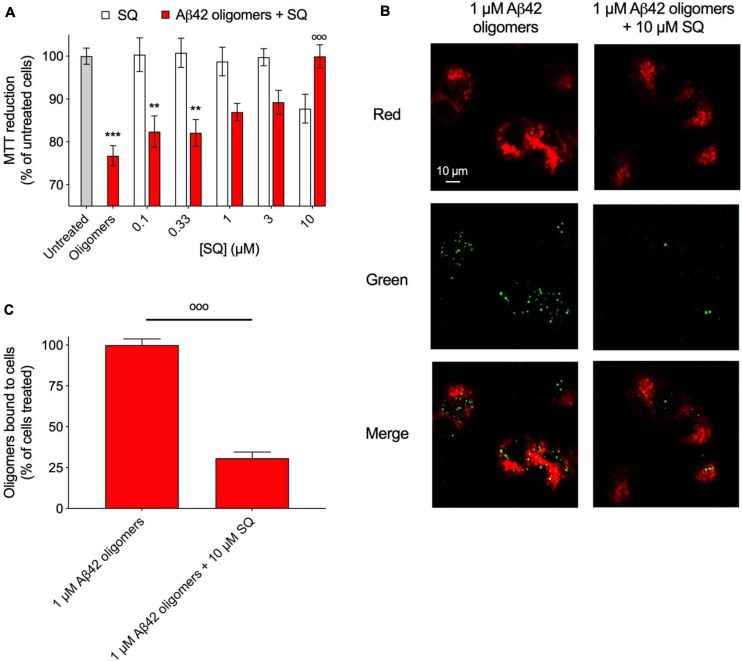
Squalamine suppresses the toxicity associated with Aβ_42_ oligomeric species to neuroblastoma cells. **(A)** Aβ-derived diffusible ligands (ADDLs) of Aβ_42_ (denoted Aβ_42_ oligomers) were resuspended in cell culture medium at a concentration of 1 μM (in monomer equivalents) and incubated with or without increasing concentrations (0.1, 0.33, 1, 3, and 10 μM) of SQ (red bars) for 1 h at 37°C and then added to the cell culture medium of SH-SY5Y cells for 24 h. The cells were also treated with the same concentrations of SQ pre-incubated in the absence of oligomers for 1 h at 37°C (white bars). **(B)** Representative confocal scanning microscopy images of the apical planes of cells treated for 15 min with Aβ_42_ oligomers (1 μM in monomer equivalents) in the absence or presence of 10 μM SQ. Red and green fluorescence indicates the cell membranes and the Aβ_42_ oligomers, respectively. Scale bar = 10 μm. **(C)** The histogram shows the percentage of colocalization on regions of interest (12–22 cells). In all panels, data represent mean ± s.e.m. of three independent experiments, the symbols ** and *** indicate *p* < 0.01 and 0.001, respectively, relative to untreated cells, and the symbol °°° indicates *p* < 0.001 relative to cells treated with oligomers. 3-(4,5-Dimethylthiazol-2-yl)-2,5-diphenyltetrazolium bromide (MTT) data were analyzed by one-way ANOVA followed by Bonferroni’s post comparison test. Cell binding data were analyzed using an unpaired, two-tailed Student’s *t*-test.

Following the above observations that SQ and its derivatives exerted similar modifications to the kinetics of aggregation and the physicochemical properties of oligomers in comparison with TRO, previous observations that SQ displaces αS oligomers from cell membranes (Perni et al., 2017), and considering that cellular assays have significantly lower throughput than the aforementioned biophysical and kinetic experiments, we sought to determine if SQ also possessed the ability to displace toxic oligomers of previously unexplored proteins from multiple protein sources from cell membranes, thereby reducing the deleterious effects of these aggregates. To begin, we first explored the impact of SQ treatment for 24 h at 37°C on SH-SY5Y neuroblastoma cells using the MTT reduction assay, which assesses the viability of cells (Perni et al., 2017). In our previous study, we found that SQ did not significantly change the viability of cells up to the maximum tested concentration of 3 μM (Perni et al., 2017). In direct agreement with our previous results, these new measurements indicate that SQ treatment does not change the overall health of the cells at concentrations equal to or below 3 μM, as seen by the MTT reduction relative to untreated cells. However, we observed that cell viability was decreased by 12 ± 4% upon the exposure of cells to 10 μM SQ (Figure 6A), thus, highlighting the physiological range of SQ in these cells and that concentration of SQ at and above 10 μM can cause small, but relevant reductions in cell health.

Next, Aβ_42_ oligomers (ADDLs) were prepared and incubated (1 μM in monomer equivalents) with increasing concentrations of SQ (0.1 to 10 μM) for 1 h at 37°C in a cell culture medium and then incubated with SH-SY5Y neuroblastoma cells for 24 h at 37°C. Viability was measured using the aforementioned MTT assay, and a dose-dependent decrease in oligomer toxicity was observed with increasing concentrations of SQ (Figure 6A). The fact that 10 μM SQ alone decreased cell health by 12 ± 4%, whereas cells treated with both 10 μM SQ and oligomers had a viability level of 100 ± 3% relative to untreated cells is likely related to the binding of SQ to the oligomers, which would reduce the effective concentration of SQ delivered to the cells.

In light of the similarities between SQ and TRO in ameliorating the toxicity caused by protein misfolding oligomers to human neuroblastoma cells, we next explored the quantity of aggregates which were able to interact with cell membranes in the absence and presence of the SQ, as previously described (Perni et al., 2017). Confocal microscopy experiments were employed to measure oligomer binding to apical planes of cell membranes in the presence of 10 μM SQ, for which a decrease in aggregate binding by 69 ± 4% was observed (Figures 6B,C). We refer to this effect as a displacement as SQ was originally shown to displace monomeric and oligomer forms of αS from the membranes of vesicles and cells, respectively (Perni et al., 2017). Moreover, the similar TRO compound was shown to bind to the membrane with high affinity (<500 nM) and make it refractory to the binding of the oligomers (Errico et al., 2020). It is, therefore, likely that aminosterols function by displacing oligomers and by preventing their association with cell membranes prior to oligomer binding. Collectively, the MTT and binding results are highly similar as those obtained for TRO, with the exception that 10 times more molecule was necessary in the case of SQ to observe comparable effects to TRO from our previous publication for cell viability experiments and measurements on the extent of oligomer binding to cell membranes (Limbocker et al., 2019). These results are therefore similar mechanistically to those obtained for TRO in the presence of multiple types of oligomers (Limbocker et al., 2020b).

**FIGURE 7:**
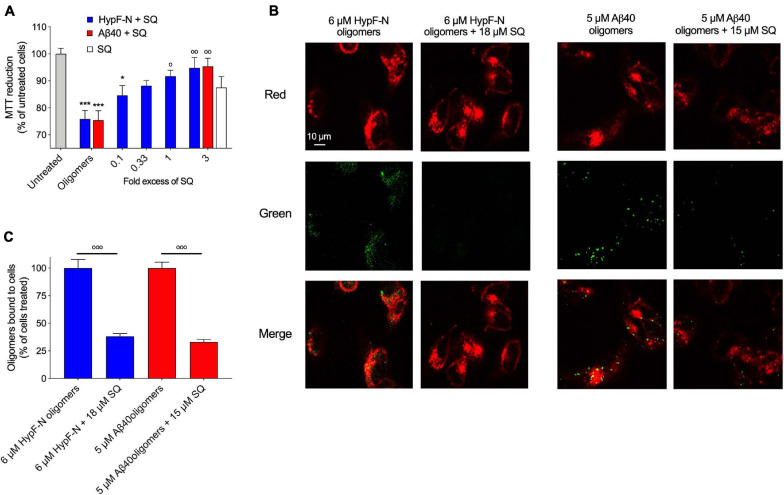
Squalamine reduces the membrane binding affinity and related toxicity of Aβ_40_ stabilized by Zn^2+^ and HypF-N oligomers to cultured human neuroblastoma cells. **(A)** HypF-N oligomers (6 μM in monomer equivalents) were resuspended in cell culture medium in the absence and presence of 0.1, 0.33, 1, and 3 molar equivalents of SQ (blue), incubated (1 h, 37°C), and subsequently added to the cell culture medium of SH-SY5Y cells for 24 h. Cells were treated under the same conditions with 18 μM SQ in the absence of oligomers (white bar). Similarly, Zn^2+^-stabilized Aβ_40_ oligomers (5 μM in monomer equivalents) were resuspended in cell culture medium in the absence and presence of a 1:3 ratio of Aβ_40_-to-SQ (red). **(B)** Representative confocal scanning microscopy images of the apical sections of SH-SY5Y cells treated for 15 min with HypF-N oligomers (6 μM in monomer equivalents, left panels) and Zn^2+^-stabilized Aβ_40_ oligomers (5 μM in monomer equivalents, right panels) in the absence and presence of a 1:3 ratio of oligomers-to-SQ. Red and green fluorescence indicates the cell membranes and the oligomers, respectively. Scale bar = 10 μm. **(C)** Histograms show the percentage of colocalization between membranes and oligomers in the regions of interest (12–22 cells in total). In all panels, data represent mean ± s.e.m. of three independent experiments, the symbols * and *** indicate *p* < 0.05 and 0.001, respectively, relative to untreated cells, and the symbols °, °°, and °°° indicate *p* < 0.05, 0.01, and 0.001, respectively, relative to cells treated with oligomers. MTT data were analyzed by one-way ANOVA followed by Bonferroni’s post comparison test. Cell binding data were analyzed using an unpaired, two-tailed Student’s *t*-test.

We then sought to determine if the observation that greater concentrations of SQ were necessary to observe the same effect as a lesser amount of TRO could be validated by studying the toxicity and membrane interaction affinity of HypF-N and Zn^2+^-stabilized Aβ_40_ oligomers in the absence and presence of SQ. In a highly similar manner as described for Aβ_42_ oligomers, MTT measurements were carried out with a pre-incubation step of 1 h at 37°C for HypF-N oligomers (6 μM in monomer equivalents) in the presence of increasing concentrations of SQ (0.1-, 0.33-, 1-, and 3-fold excesses). Similar to as described above, we observed that cell viability was decreased by 13 ± 5% upon the exposure of cells to 18 μM SQ in the absence of oligomers (Figure 7A), further confirming that concentrations of SQ at and above 10 μM can cause small decreases in cell health. We next observed that this aminosterol induced a dose-dependent decrease in HypF-N oligomer toxicity. We also measured the toxicity of the Aβ_40_ oligomers (5 μM in monomer equivalents) in the presence of a threefold excess of SQ, for which a near-complete attenuation of toxicity was revealed. Indeed, the toxicities for both types of oligomers in the presence of a threefold excess of SQ was not significantly different than the toxicity resulting from the molecule alone (Figure 7A), suggesting that the oligomers are largely inert in terms of their ability to induce cellular dysfunction under these conditions. Oligomer toxicity has been previously observed to plateau at a concentration of approximately 6 μM for HypF-N oligomers and 5 μM for Zn^2+^-stabilized Aβ_40_ oligomers (Limbocker et al., 2020b), which is broadly consistent with the oligomer toxicities observed here. Given that greater concentrations of oligomers for HypF-N and Zn^2+^-stabilized Aβ_40_ were required in our measurements to observe a comparable decrease in cell viability with respect to only 1 μM Aβ_42_ oligomers, it was not pragmatic to explore ratios greater than a threefold excess of SQ-to-oligomers for HypF-N and Aβ_40_ oligomers due to the intrinsic toxicity of SQ above 10 μM (Figures 6A, 7A).

**FIGURE 8:**
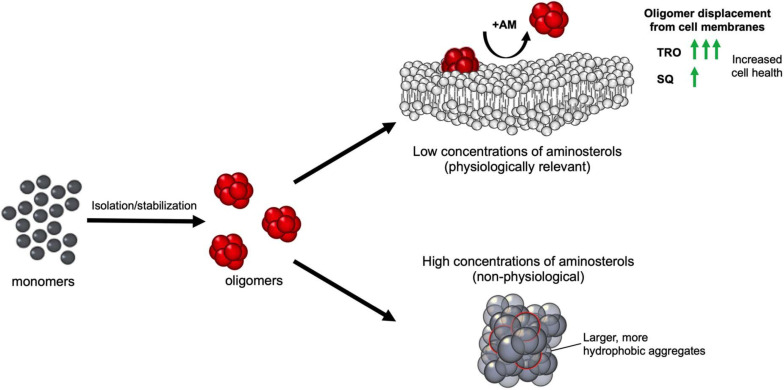
Schematic representation of the effect of aminosterols on isolated or stabilized oligomers of αS, Aβ_40_ stabilized by Zn^2+^, and HypF-N. After oligomer isolation or stabilization, the addition of aminosterols (AM) to the reaction mixture can induce the non-physiological clustering of the aggregates at high concentrations (shown as gray species), and the physiologically relevant displacement of oligomers from cell membranes at low concentrations (toxic oligomers are shown as red species). For the latter scenario, trodusquemine exhibited comparable effects with squalamine at 3–10× lower concentrations of the molecule, signaling its enhanced efficacy at displacing protein misfolded oligomers from cell membranes.

Finally, HypF-N and Aβ_40_ oligomers (6 and 5 μM, in monomer equivalents, respectively) were incubated with cells for 15 min as previous described in the absence and presence of a threefold excess of SQ. Confocal microscopy was employed as previously described (Perni et al., 2017) to measure the extent of the interaction of the oligomers with the cellular membrane. Extensive oligomer binding was observed in the absence of the molecule, but in its presence, the bound fraction was dramatically reduced (Figure 7B), as only 33 ± 2% of Aβ_40_ oligomers and 38 ± 3% of HypF-N oligomers were localized to neuroblastoma cell membranes in the presence of a threefold excess of SQ relative to cells treated with oligomers alone (Figure 7C). We, therefore, conclude that SQ and TRO are equivalent in their mechanism of function with respect to the suppression of oligomer toxicity via their displacement from cell membranes, with a major difference arising from the observation that three times greater concentrations of SQ for these protein systems were needed to observe comparable effects in comparison with TRO from Limbocker et al. (2020b). Therefore, SQ appears to be equally effective in these cellular assays at displacing oligomers and preventing their toxicity relative to TRO; however, a three- to 10-fold greater concentration of SQ was needed to observe the same effects for TRO, dependent upon the proteinaceous composition of oligomeric species under investigation (Figures 6–8).

## Discussion

The results that we have presented extend to SQ the previous conclusion obtained with TRO (Perni et al., 2017, 2018; Limbocker et al., 2019, 2020b) that aminosterols can outcompete oligomers and induce the displacement of these cytotoxic aggregates of multiple protein sources from cell membranes, thereby suppressing the damage caused by protein aggregation. At low TRO concentrations, TRO seems to bind to the membrane, not oligomers (Limbocker et al., 2020b). At high TRO concentrations, there is an excess of TRO, and binding is also observed in the oligomers in addition to the membrane (Limbocker et al., 2020a). At small TRO concentrations, binding to the membrane prevails. In contrast, the effects of these compounds on the overall aggregation reactions were found to be different on the two proteins, Aβ_42_ and αS, that we studied. Similar to our previous finding on TRO (Limbocker et al., 2019), SQ targeted and enhanced the rate of Aβ_42_ aggregation by potentiating mostly the secondary nucleation microscopic step to produce shorter and wider fibrils, which likely also hastens the conversion of more harmful oligomers to relatively inert fibrillar aggregates. αS was targeted differently by these aminosterols, as they inhibited its aggregation by suppressing lipid-induced nucleation, as found here for SQ and its derivatives and previously with TRO (Perni et al., 2018). In a previous report, it was also found that the fibril amplification of αS was also inhibited by TRO (Perni et al., 2018), although this microscopic step was not studied with SQ. Both this inhibition on αS aggregation and acceleration on Aβ_42_ fibril formation can reduce oligomer toxicity by decreasing the number of toxic species produced over time. In addition to modulating the aggregation kinetics in modes that may work to suppress the populations of toxic species, we further observed that the aminosterols directly displace such toxic species from cell membranes. While both aminosterols were efficacious in reducing toxicity, TRO appears to have a higher efficacy than SQ given its ability to achieve comparable protective levels at 3–10 times less molecules, which may result from the differing polyamine structures fused to the sterol rings.

For stabilized oligomers, we have shown previously that the predominant means by which TRO protects cells from stabilized oligomers of αS, Aβ_40_, and HypF-N is through their displacement from cell membranes, rather than a change in oligomer structure at physiologically relevant concentrations (Limbocker et al., 2020b). This results from the ability of TRO to bind with high affinity (<500 nM) to the hydrophilic portion of the phospholipid bilayer. In particular, this small molecule was found to localize within the external hydrophilic layer down to the interface between the hydrophilic and hydrophobic layers with a well-defined angle of ca. 55° for the major axis of the molecule with respect to the normal to the bilayer plane and a superficial positioning of the positively charged spermine moiety (Errico et al., 2020). This causes a decrease in its negative charge, its reinforcement against oligomer penetration, and a redistribution of its cholesterol and ganglioside GM1 molecules, all known to reinforce the membrane against the deleterious action of misfolded protein oligomers (Errico et al., 2020). The increase in oligomer size observed herein upon SQ addition is expected to reduce oligomer toxicity toward cells, while the increase in oligomer hydrophobicity would be expected to increase the ability of these species to interact with cell membranes and induce dysfunction (Mannini et al., 2014; Cappelli et al., 2016; Limbocker et al., 2020a). The increase in both size and hydrophobicity would, therefore, be expected to offset each other in terms of their effects on oligomer cytotoxicity, as was shown recently with very similar size–hydrophobicity relationships using increasing concentrations of rationally designed antibodies and Aβ_40_ oligomers (Limbocker et al., 2020a). However, this effect is not likely to be physiological given the high aminosterol concentration regimes used to observe these physicochemical changes *in vitro*. These results obtained here with SQ, therefore, agree with our previous conclusion that the dominant mode by which TRO prevents oligomer toxicity is by their displacement from cell membranes.

As seminal agents in numerous protein misfolding diseases, we focused in this report on the misfolded oligomers formed by these proteins rather than on other conformers. We note, however, that aminosterols have been shown to bind to amyloid fibrils (Perni et al., 2017, 2018), resulting in the enhancement of monomer-dependent secondary nucleation for Aβ_42_ (Limbocker et al., 2019) and the inhibition of fibril amplification for αS (Perni et al., 2018). Squalamine can bind monomeric αS at high concentrations, causing small chemical shifts in the C-terminal region of the protein (Perni et al., 2017); however, trodusquemine was found not to impact the normal association of monomeric αS with DMPS vesicles at physiological concentrations (Perni et al., 2018). The detailed molecular mechanism by which aminosterols bind these different proteins and conformers is an important topic for future work that has key implications for the rate of oligomer formation during the aforementioned processes and could explain why Aβ_42_ aggregation is enhanced and αS nucleation reduced by aminosterols.

Relative to the other aminosterols, we observed that desSQ modulates the aggregation of Aβ_42_ and αS to a lesser extent (Figure 4). Similarly, desSQ demonstrated a reduced ability to modify the size of stabilized oligomers (Figure 5 and Supplementary Figures 3–5). Collectively, these data suggest that the anionic group in this position on the sterol side chain (Figure 1) may be important to its activity. As the behavior of αSQ and SQ were highly similar across all our measurements, these data further suggest that the chirality of the polyamine with respect to the sterol may not be essential to its activity.

The difference in effectiveness between TRO and SQ points toward slight differences in structure that may highlight the framework for a rational approach toward identifying and developing countermeasures for differing neurodegenerative diseases. TRO and SQ have spermine and spermidine polyamine side chains, respectively, and they are both small molecules with a hydrophobic sterol domain that can integrate into cell membranes. Moreover, the aminosterol derivatives differ in their carbon skeletons and the distribution of their functional groups. The difference of one positive charge between SQ and TRO in its side chain appeared to have a significant impact on the respective efficacies of these aminosterols, with the more charged TRO having an apparently 3–10 times greater protective power relative to an equivalent concentration of SQ. This argument is supported by the observation that TRO decreases the negative charge of the membrane, as determined with zeta-potential measurements, and neutralizes in part the negative moieties of the ganglioside GM1 (Errico et al., 2020), known to mediate oligomer–membrane interaction and oligomer toxicity (Evangelisti et al., 2016). A less positively charged SQ very likely can only be less effective in causing this neutralization. Additionally, TRO has been reported to be able to cross the blood–brain barrier, whereas SQ has not (Lantz et al., 2010), which could potentially impact the utility of these molecules in clinical trials against neurodegenerative disease. These results also point to the potential of other members of the aminosterol family that could be synthesized in the future, which may be differentially efficacious based on similar alterations in structure and electrostatic interactions.

Collectively, these results highlight that small alterations in the structure of squalamine derivatives can alter their effectiveness against the toxic oligomers causing protein misfolding diseases. Moreover, our research highlights the differences between the aggregation processes in AD and PD, resulting in unique responses to the same aminosterols. Despite the differences in the aggregation reactions of these proteins at a fundamental level, both SQ and TRO appear to be effective in decreasing the concentration of cytotoxic protein aggregates of both proteins. In addition, they were effective in counteracting the cytotoxicity of both protein systems, demonstrating that membrane displacement is likely to be an effective strategy for combatting multiple protein misfolding diseases. These results, along with the observed differences in molecule effectiveness, point toward a rational framework for the identification and development of small molecule therapeutics that address oligomer cytotoxicity through membrane displacement.

## Data Availability Statement

The raw data supporting the conclusions of this article will be made available by the authors, without undue reservation.

## Author Contributions

RL, RS, SC, FR, BM, CX, MP, RC, GM, JH, PF, JK, CC, DB, MZ, TPJK, CD, FC, and MV designed the research. RL, RS, SC, FR, BM, CX, RC, AB, TK, and JK performed the research. RL, RS, SC, FR, BM, CX, RC, and GM analyzed the data. RL, RC, LS, NB, AW, RK, EC, SE, JEH, LN, KL, CC, FC, and MV contributed to the first draft of the manuscript. RL, FC, and MV supervised the study. All authors were involved in the editing of the manuscript.

## Disclaimer

The views expressed herein are those of the authors and do not reflect the position of the United States Military Academy, the Department of the Army, or the Department of Defense.

## Conflict of Interest

DB and MZ are inventors in a patent for the use of aminosterols in the treatment of Parkinson’s disease. DB and MZ are co-founders of Enterin Inc. and serve as the President and CSO, respectively, of the company. MV, TPJK, and JH are co-founders, and BM and MP are employees of Wren Therapeutics Ltd., which is independently pursuing inhibitors of protein misfolding and aggregation. The remaining authors declare that the research was conducted in the absence of any commercial or financial relationships that could be construed as a potential conflict of interest.

## Supplementary Material

Supplementary Figure 1 Raw ThT data for the Aβ_42_ aggregation experiments in the presence of increasing concentrations of aminosterols.

Supplementary Figure 2 Squalamine modifies the aggregation of Aβ_42_ in a similar manner as trodusquemine.

Supplementary Figure 3 Aminosterols increase the size and hydrophobicity of αS oligomers.

Supplementary Figure 4 Aminosterols increase the size and hydrophobicity of HypF-N oligomers.

Supplementary Figure 5 Aminosterols increase the size and hydrophobicity of Aβ_40_ oligomers stabilized by Zn^2+^.
