# Supplementary material for: Squalamine and Its Derivatives Modulate the Aggregation of Amyloid-β and α-Synuclein and Suppress the Toxicity of Their Oligomers
Source: Front Neurosci. 2021 Jun 18;15:680026. doi: 10.3389/fnins.2021.680026 (PMC8249941; doi:10.3389/fnins.2021.680026)
Supplement: Supplementary Figure 1 — Raw ThT data for the Aβ42 aggregation experiments in the presence of increasing concentrations of aminosterols. [file Data_Sheet_1.PDF]

Supplementary Material

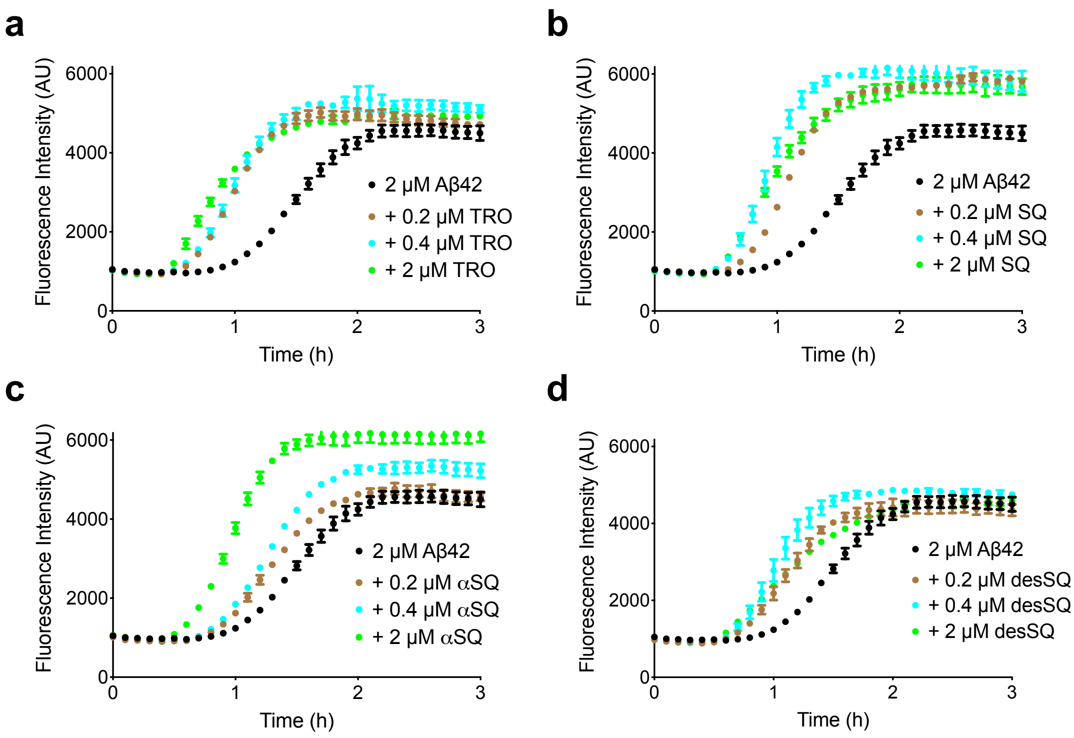

Figure S1. Raw ThT data for the Aβ_{42} aggregation experiments in the presence of increasing concentrations of aminosterols. Kinetic profiles of the aggregation of 2 µM Aβ_{42} in the absence (black) or presence of 0.2 µM (brown), 0.4 µM (cyan) and 2 µM (green) of TRO (**a**), SQ (**b**), αSQ (**c**) and desSQ (**d**). Data represent mean ± standard error of the mean (s.e.m.) of three technical replicates.

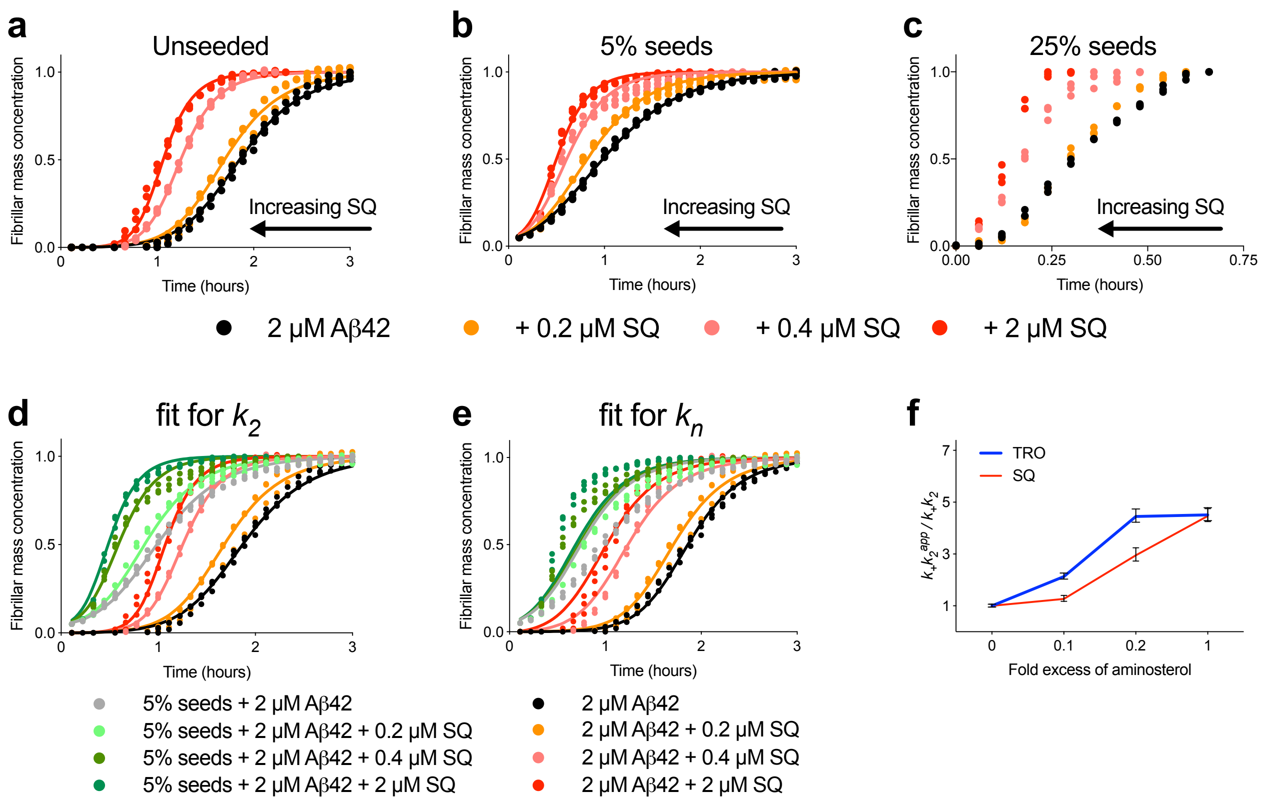

Figure S2. Squalamine modifies the aggregation of Aβ_{42} in a similar manner as trodusquemine. (**a**) Kinetic profiles of the aggregation of 2 μM Aβ_{42} in the absence (black) or presence of squalamine at ratios of Aβ42:squalamine of 10:1, 5:1, and 1:1 (light to dark red, ranging from 0.2 to 2 μM). Solid lines indicate theoretical predictions based on kinetic fitting (see Results); data are well-descried by varying the rates associated with just the secondary nucleation pathway (k_+k_2). (**b**) Kinetic profiles of the aggregation of 2 μM Aβ_{42} in the presence of 5% fibril seeds and in the absence (black) or presence of squalamine at ratios of 10:1, 5:1, and 1:1 (light to dark red). Solid lines indicate theoretical predictions based on kinetic fitting (see Results); data are well-descried by varying the rates associated with just monomer-dependent secondary nucleation (k_2). (**c**) Kinetic profiles of the aggregation of 2 μM Aβ_{42} in the presence of 25% fibril seeds and in the absence (black) or presence of squalamine at ratios of 10:1, 5:1, and 1:1 (light to dark red). (**d**) As was observed for trodusquemine, the unseeded and 5% seeded data were well-described for the global modification of k_2, while global fits for k_n were poor (**e**). (**f**) Dependency of the apparent reaction rate constants of secondary pathways (k_+k_2) on increasing concentrations of squalamine (red). Data represent mean ± s.e.m. of three technical replicates. Shown for comparison are the rates for trodusquemine (blue, previously published in Limbocker et al., 2019). These measurements were carried out alongside a comparison of the effect of trodusquemine on Aβ_{42} aggregation, and the aggregation traces in the absence of squalamine therefore are the same data as previously published (Limbocker et al., 2019).

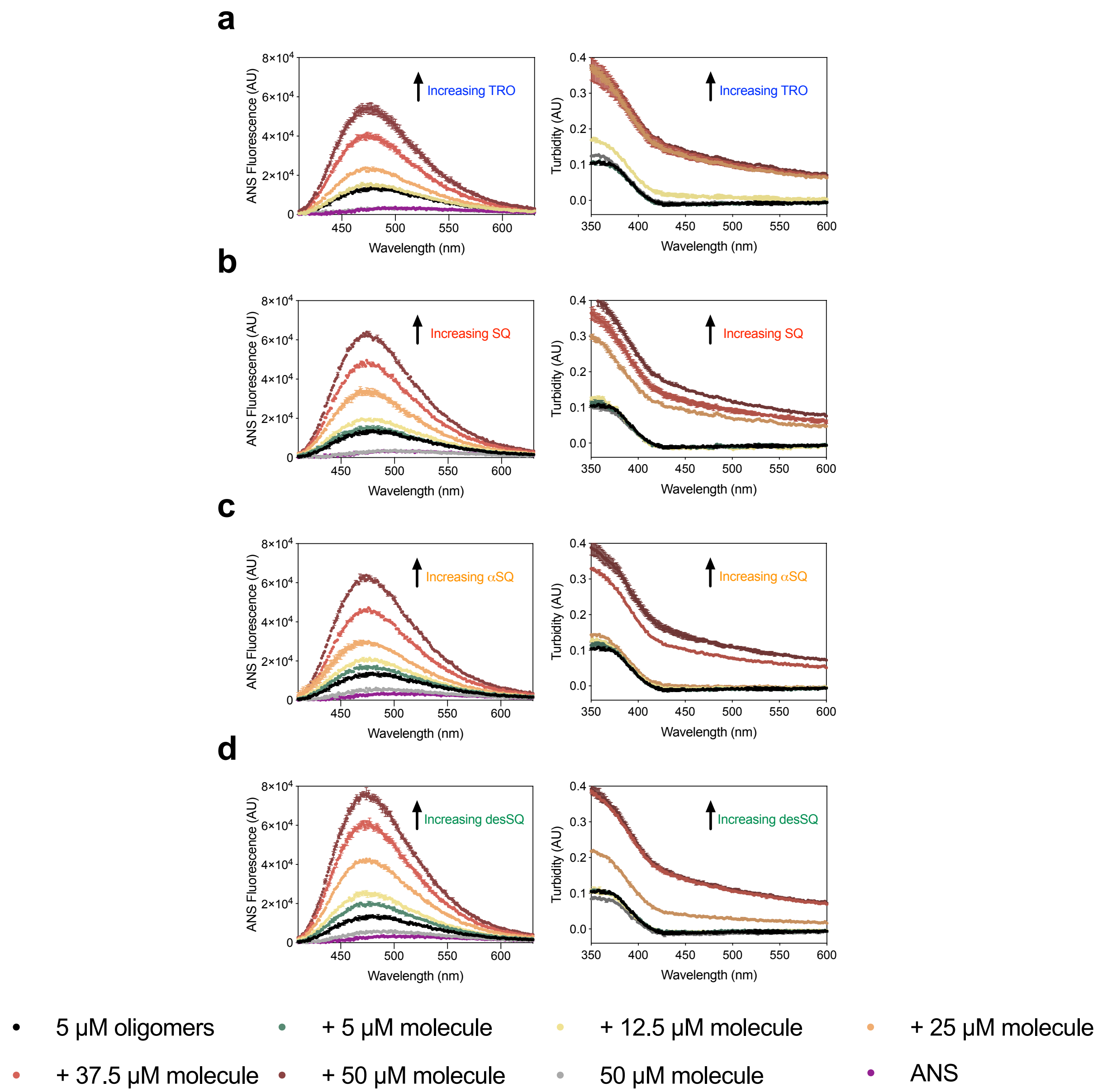

Figure S3. Aminosterols increase the size and hydrophobicity of αS oligomers. The effects of aminosterols (0-10 fold excesses, represented in different colors) on the physicochemical properties of oligomers comprised of αS were monitored using ANS binding (left panels) and turbidity absorbance (right panels) to measure hydrophobicity and size, respectively, for trodusquemine (**a**; blue), squalamine (**b**; red), αSQ (**c**; orange) and desSQ (**d**; green). Measurements of ANS in the absence (purple traces) and presence of 50 μM of each molecule (grey traces), corresponding to the highest concentration tested in vitro, are shown for reference. Data represent mean ± s.e.m. of two technical replicates.

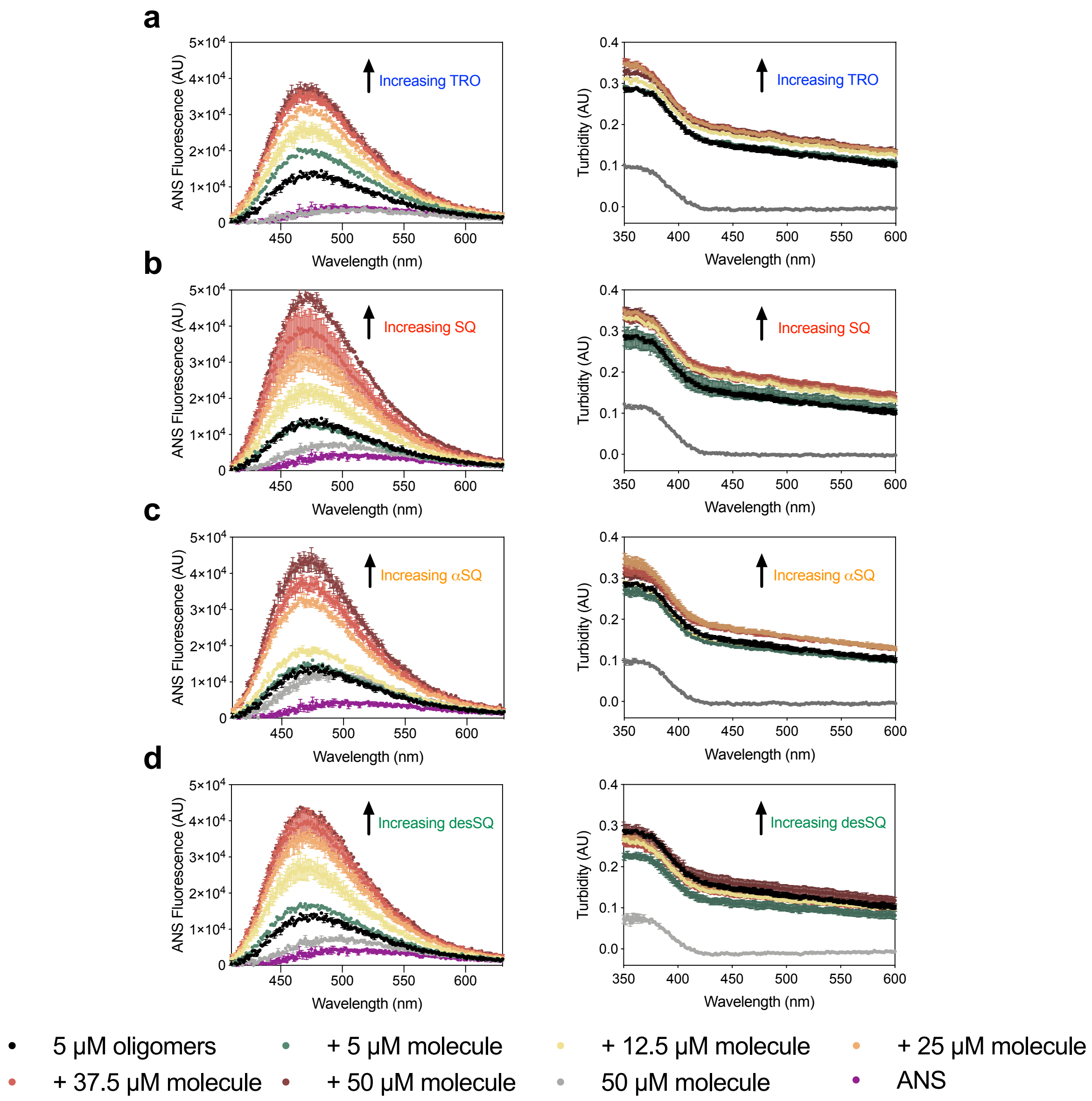

Figure S4. Aminosterols increase the size and hydrophobicity of HypF-N oligomers. The effects of aminosterols (0-10 fold excesses, represented in different colors) on the physicochemical properties of oligomers were monitored using ANS binding (left panels) and turbidity absorbance (right panels) to measure hydrophobicity and size, respectively, for trodusquemine (**a**; blue), squalamine (**b**; red), αSQ (**c**; orange) and desSQ (**d**; green). Measurements of ANS in the absence (purple traces) and presence of 50 µM of each molecule (grey traces), corresponding to the highest concentration tested in vitro, are shown for reference. Data represent mean ± s.e.m. of two technical replicates.

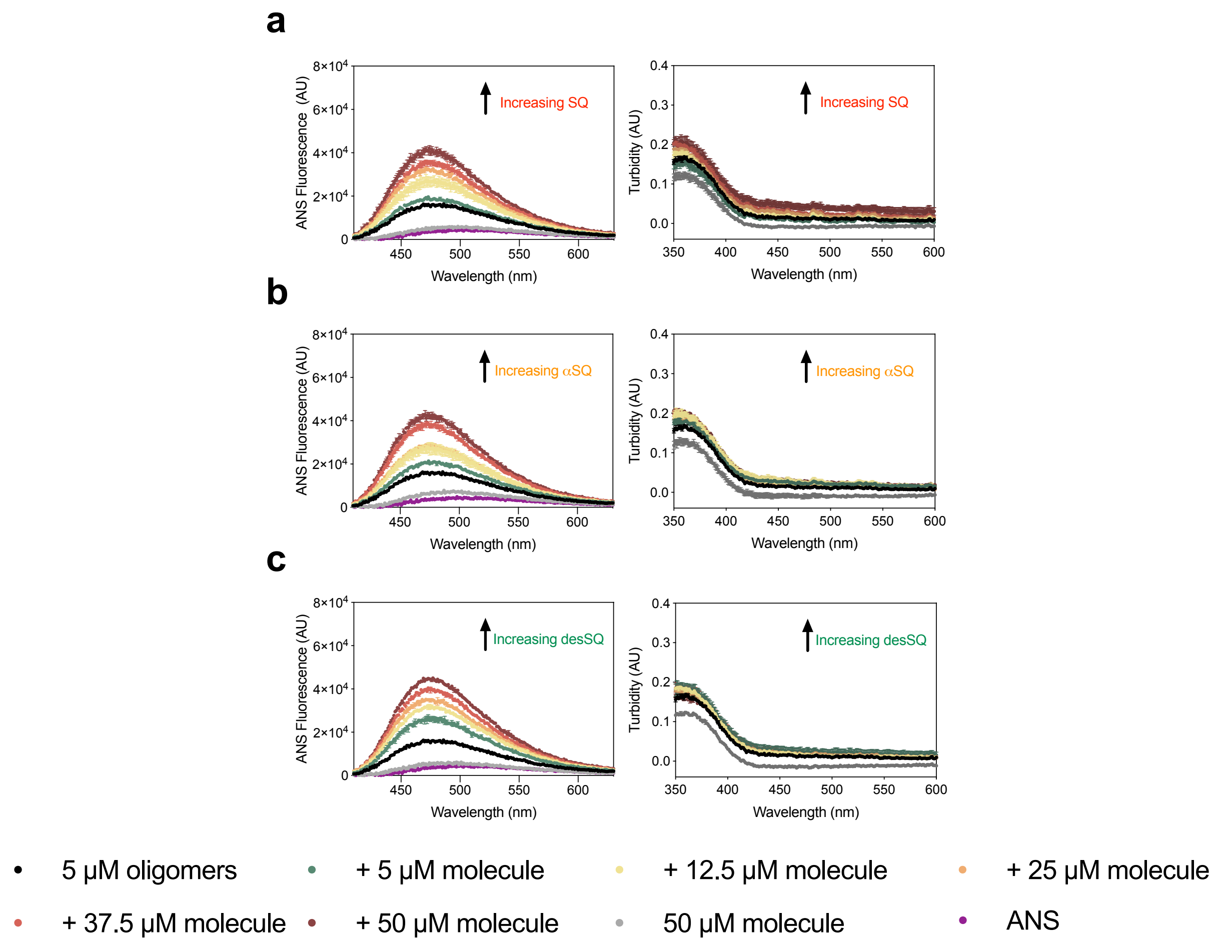

Figure S5. Aminosterols increase the size and hydrophobicity of $A\beta_{40}$ stabilized by Zn^{2+}. The effects of aminosterols (0-10 fold excesses, represented in different colors) on the physicochemical properties of oligomers comprised of $A\beta_{40}$ were monitored using ANS binding (left panels) and turbidity absorbance (right panels) to measure hydrophobicity and size, respectively, for squalamine (**a**; red), αSQ (**b**; orange) and desSQ (**c**; green). Measurements of ANS in the absence (purple traces) and presence of 50 μM of each molecule (grey traces), corresponding to the highest concentration tested in vitro, are shown for reference. Data represent mean ± s.e.m. of two technical replicates. These measurements were carried out alongside a comparison of the effect of trodusquemine on $A\beta_{40}$ oligomers, for which highly similar results as those observed here with these three aminosterols were obtained (Limbocker et al., IJMS, 2020a).
